# Using parallelized incremental meta-docking can solve the conformational sampling issue when docking large ligands to proteins

**DOI:** 10.1186/s12860-019-0218-z

**Published:** 2019-09-05

**Authors:** Didier Devaurs, Dinler A Antunes, Sarah Hall-Swan, Nicole Mitchell, Mark Moll, Gregory Lizée, Lydia E Kavraki

**Affiliations:** 1 Department of Computer Science, Rice University, 6100 Main St, Houston, TX 77005 USA; 2 Department of Melanoma Medical Oncology - Research, The University of Texas MD Anderson Cancer Center, 1515 Holcombe Blvd, Houston, TX 77030 USA

**Keywords:** Molecular docking, Protein-ligand docking, Protein-peptide docking, Conformational sampling, Scoring, Parallelism, Incremental protocol

## Abstract

**Background:**

Docking large ligands, and especially peptides, to protein receptors is still considered a challenge in computational structural biology. Besides the issue of accurately scoring the binding modes of a protein-ligand complex produced by a molecular docking tool, the conformational sampling of a large ligand is also often considered a challenge because of its underlying combinatorial complexity. In this study, we evaluate the impact of using parallelized and incremental paradigms on the accuracy and performance of conformational sampling when docking large ligands. We use five datasets of protein-ligand complexes involving ligands that could not be accurately docked by classical protein-ligand docking tools in previous similar studies.

**Results:**

Our computational evaluation shows that simply increasing the amount of conformational sampling performed by a protein-ligand docking tool, such as Vina, by running it for longer is rarely beneficial. Instead, it is more efficient and advantageous to run several short instances of this docking tool in parallel and group their results together, in a straightforward parallelized docking protocol. Even greater accuracy and efficiency are achieved by our parallelized incremental meta-docking tool, DINC, showing the additional benefits of its incremental paradigm. Using DINC, we could accurately reproduce the vast majority of the protein-ligand complexes we considered.

**Conclusions:**

Our study suggests that, even when trying to dock large ligands to proteins, the conformational sampling of the ligand should no longer be considered an issue, as simple docking protocols using existing tools can solve it. Therefore, scoring should currently be regarded as the biggest unmet challenge in molecular docking.

## Background

One of the most important biomedical applications of structural biology is drug discovery [1–5]. Proteins are essential components of living cells, performing structural functions, chemical reactions, transportation, signaling, and so on. Most of these functions involve molecular interactions with other proteins, nucleic acids or small molecules (i.e., ligands or peptides). The study of protein-ligand interactions is key to understanding molecular pathways, which in turn can provide opportunities for diagnosis and treatment of pathological conditions (e.g., using a drug to inhibit a key enzyme). Computational tools play a central role in this field [2]. In particular, molecular docking tools are routinely used to predict the most likely binding mode between a ligand and a protein receptor (which is often referred to as *geometry optimization*), or to screen thousands of ligands in search of potential binders to a target protein (which is referred to as *virtual screening*) [6, 7].

At the core of every molecular docking method lie *sampling* and *scoring* [8–10]. The first component, conformational sampling, relates to the challenge of exploring ligand flexibility. Most molecules involve rotatable bonds allowing them to adopt alternative conformations in solution. Furthermore, the number of possible conformations increases exponentially with the number of rotatable bonds. This underpins the computational challenge of exploring all these conformations and predicting the best fit between a ligand and a protein’s binding site [11]. With the exception of very small ligands, exhaustively exploring all the rotatable bonds—or degrees of freedom (DoFs)—of a ligand is infeasible, and several strategies have been proposed to achieve efficient sampling [8, 12, 13].

Other considerations that we do not address in this paper can render conformational sampling even more computationally challenging. This is the case when considering the flexibility of the protein receptor in addition to that of the ligand. As there might exist structural differences between a protein’s bound and unbound conformations, ideally, protein flexibility should be taken into account in molecular docking studies. However, due to the tremendous computational cost of doing so, various methods have been proposed that consider only limited levels of flexibility [6]. Another important issue is the inclusion of “explicit” water molecules in molecular docking because they sometimes mediate interactions between ligands and receptors. Unfortunately, there is currently no consensus on the way this should be done to improve the results of docking tools [9, 10, 12].

The second important component of molecular docking is scoring [9, 14]. The goal of a scoring function is to assess the “quality” of the conformations produced by the sampling algorithm in order to guide the search towards better binding modes and to rank conformations of different ligands (as in virtual screening). Since numerous conformations are evaluated during sampling, scoring functions have to be computationally efficient. This requirement imposes a major trade-off between efficiency and accuracy when designing a useful scoring function [12, 13, 15].

A variety of docking tools is now available, relying on various strategies for sampling and scoring, which both affect docking performance and involve different challenges [10, 16]. Our work has been focused on addressing the challenges associated with sampling when docking large ligands and even peptides. Our first step was to develop a parallelized incremental meta-docking approach to dock large ligands, called DINC [11, 17]. Showing promising capabilities, DINC was applied in studies on STAT3 and STAT6 inhibition [18, 19]. After significant software improvements, we demonstrated its ability to dock large peptides binding MHC receptors [20]. However, these improvements were not sufficient to ensure that DINC could accurately dock any large ligand. To address this issue, we have recently released a new version of DINC. One of the most significant changes is that DINC now mostly relies on the popular docking tool Vina [21], instead of AutoDock4 [22], as in its past versions [11, 20]. This latest upgrade has been made available through the DINC 2.0 web server [23, 24].

The rationale behind the switch from AutoDock4 to Vina is that several benchmark studies have reported that Vina performs generally better than AutoDock4. For example, in [25], it is shown that Vina outperforms AutoDock4 both in terms of sampling power and scoring power. This is especially true for the Lamarckian genetic algorithm method in AutoDock4, which is the one that was used in DINC [11, 20]. Similar differences in docking performance are reported in [26], which also illustrates the computational efficiency of Vina over AutoDock4. This confirms what was initially stated by Vina’s authors, i.e., improved scoring accuracy and sampling efficiency (through multi-threading and optimization) [21].

In this paper, we report the results of our evaluation of the sampling power of several docking protocols, including DINC. We focus on the problem of docking large ligands (including peptides) to protein receptors. For our evaluation, we use five datasets of protein-ligand complexes reported in related work and involving large ligands that could not be accurately docked using Vina or other docking tools [11, 25–28]. Although it is impossible to fully dissociate the effects of sampling and scoring on the output of a docking approach, there are approaches for assessing sampling power somewhat independently of scoring power [14, 25–28]. To do so, we check whether a given docking approach is able to produce binding modes that are similar to the crystal structure of a given complex, whether or not these binding modes receive high scores. The docking approaches we evaluate all involve Vina and are based on (i) varying Vina’s exhaustiveness (i.e., the parameter defining the amount of sampling performed by Vina), (ii) running several instances of Vina in parallel and grouping their results together, in a protocol we call Multi-Vina, and (iii) using our parallelized incremental meta-docking method, DINC.

Our results clearly show the benefits of using parallelized approaches over simply increasing Vina’s exhaustiveness. Furthermore, the good performance of DINC indicates that the incremental paradigm it relies on provides additional benefits over only using parallelism. Overall, our study suggests that, even when docking large ligands (i.e., ligands with more than a dozen DoFs), conformational sampling is rarely critical if enough computing resources are available. Although this might not be satisfactory in the context of virtual screening applications, where computational efficiency is paramount, this is evidence that the conformational sampling challenge can essentially be considered solved in the context of geometry optimization. This also highlights the fact that scoring remains the biggest unmet challenge of molecular docking.

## Results

In this section we present the results of our evaluation of several docking methodologies that involve the classical molecular docking tool Vina, including our parallelized incremental meta-docking tool, DINC. We perform redocking experiments, which consist of trying to reproduce the crystal structures of challenging protein-ligand complexes from five different datasets (see “Methods” section). The most extensive part of our benchmarking involves only the first four datasets (Dhanik, Renard, LEADS and Hou) as the fifth one (PPDbench) was published after we had performed our study. The PPDbench dataset is involved only in a smaller experiment reported at the end of this section. Note that, in our redocking tasks, we only explore the flexibility of ligands, and keep proteins rigid at all times.

To assess the quality of the results produced by a docking protocol for a specific complex, we evaluate the Root Mean Square Deviation (RMSD) between the predicted binding modes and the initial crystal structure of the complex, considering all the heavy atoms of the ligand, i.e., the so-called *all-atom RMSD*. The results we report for each complex correspond to the RMSD between its crystal structure and the so-called *top-RMSD* conformation, i.e., the conformation produced by the docking tool which is the closest to the crystal structure (see “Methods” section). This allows assessing the sampling power of a docking tool irrespective of its scoring power. Note that we consider a crystal structure to be successfully reproduced if this all-atom RMSD is less than 2 Å, which is a common threshold in the docking community.

### Vina

As explained in the “Methods” section, the protein-ligand complexes we selected for this study involve large ligands and cannot be reproduced using Vina with its default parameters. The main parameter we will refer to in this section is Vina’s exhaustiveness, which defines the amount of sampling that is performed by Vina before it returns its results, and whose default value is 8. Note that it is a unitless parameter whose value should be a positive integer, and it has no maximum value. The results we have obtained when using Vina to try and reproduce the complexes from our first four datasets are listed in Tables 1–4, under the column titled “Vina”. These results are averages (and standard deviations) calculated from five runs. For all complexes, the all-atom RMSD between the initial crystal structure and the top-RMSD conformation produced by Vina is greater than 2 Å, with an average of 5.17 Å (and a standard deviation of 1.12 Å) across the four datasets combined.

The strategy recommended by Vina’s creators to improve its performance is to increase its exhaustiveness. Therefore, we have tried to reproduce all the complexes from our first four datasets after increasing Vina’s exhaustiveness to 100. Results (averaged over five runs) are listed in Tables 1–4, under the column titled “Vina_100_”. They show limited overall improvement, with an average of 4.52 Å (and a standard deviation of 0.76 Å) across the four datasets. For some complexes, such as 4FIV in the Dhanik dataset, 2D5W in the Renard dataset, 3MMG in the LEADS dataset and 2ER6 in the Hou dataset, increasing the exhaustiveness yields a significant improvement and a successful reproduction. On the other hand, for other complexes, such as 1JQ9 in the Dhanik dataset, 1TJ9 in the Renard dataset, 3OBQ in the LEADS dataset and 1G7V in the Hou dataset, we can see a deterioration of the results. The fact that the standard deviation associated with these complexes decreased after increasing Vina’s exhaustiveness suggests that this deterioration is not due to the inherent randomness of the sampling process, but to the fact that Vina more consistently produces “bad” results.

A critical effect of increasing exhaustiveness is a rise in computing time: Vina’s runtime increases roughly linearly with respect to its exhaustiveness. Therefore, we do not report explicit running times (which depend on the computing platform) and only discuss differences in computing times through differences in exhaustiveness. In our study, increasing exhaustiveness from 8 to 100 resulted in a 12-fold increase in Vina’s running times. A legitimate question is thus whether the limited improvement in results quality achieved by increasing exhaustiveness is worth such an increase in computing time. If one was not deterred by the prohibitive running times, one could increase exhaustiveness beyond 100 and hopefully get better results. However, our experience and other studies have shown that, when docking large ligands, increasing Vina’s exhaustiveness only results in minor improvements that quickly plateau [26, 27].

### Multi-Vina

We aimed to make better use of computing resources than is achieved by increasing Vina’s exhaustiveness. To that end, we assessed a docking protocol we call “Multi-Vina”, based on running several independent instances of Vina (performing different non-deterministic conformational searches) in parallel and grouping their results together. This method generates a larger pool of binding modes from which we extract the top-RMSD conformation.

**Fig. 1:**
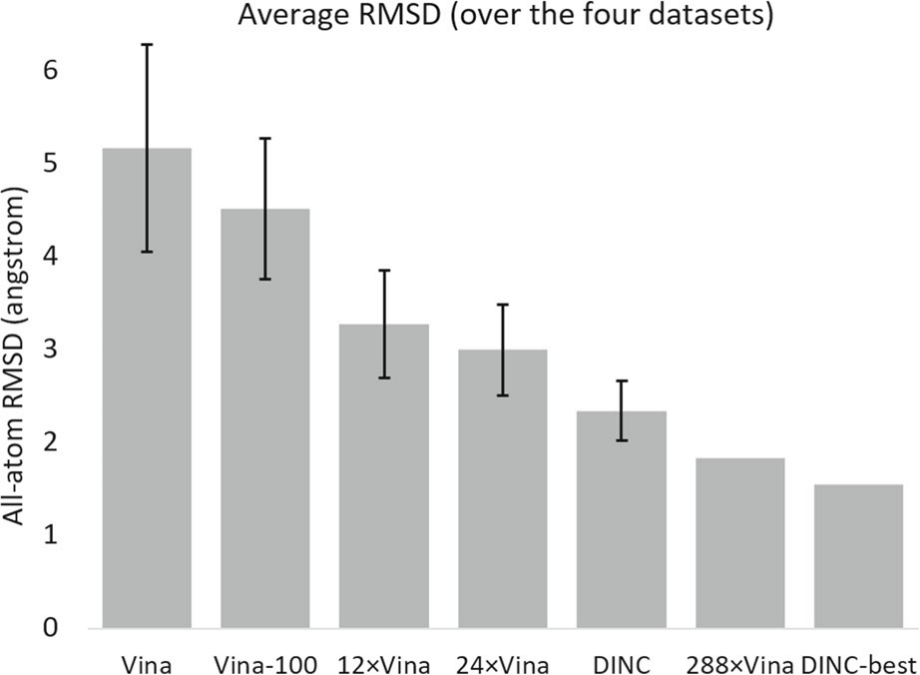
Average RMSD achieved by the docking protocols. For each docking protocol, we report the all-atom RMSD averaged over all complexes from the first four datasets. For Vina, Vina_100_, 12 ×Vina, 24 ×Vina and DINC, we also report the corresponding standard deviation

The first protocol we evaluated involves running 12 instances of Vina, with its exhaustiveness set to 8; we call it 12 ×Vina. Using 12 ×Vina requires as much computing resources as using Vina_100_ (in terms of CPU time), but as little time as running Vina (in terms of wall clock time). The results (averaged over five replicates) we obtained when trying to redock the complexes from our first four datasets with this protocol are listed in Tables 1–4, under the column titled “12 ×Vina”. The overall average and standard deviation across the four datasets are 3.28 Å and 0.58 Å, respectively. As expected, 12 ×Vina performs better than Vina, and interestingly it also performs significantly better than Vina_100_ (see Fig. 1) despite using a similar amount of computing resources. The only complexes for which Vina_100_ produced a better result than 12 ×Vina are 1N12 and 1H6W in the LEADS dataset, as well as 3FVH in the Hou dataset.

To assess the impact of increasing the amount of computing resources allocated to Multi-Vina, we also tried to redock the complexes from our first four datasets using a 24 ×Vina protocol. Results (averaged over five replicates) are listed in Tables 1–4, under the column titled “24 ×Vina”. The overall average and standard deviation across the four datasets are 3 Å and 0.49 Å, respectively. This is only a small improvement when compared to results obtained with the 12 ×Vina protocol (see Fig. 1), especially considering that computing resources have been doubled.

Finally, to evaluate the full potential of the Multi-Vina method, we performed redocking experiments with a 288 ×Vina protocol (where 288=12×24) on our first four datasets. However, because of the huge amount of computing resources required and the very low expected standard deviation, we performed only one replicate for this experiment. Results are listed in Tables 1–4, under the column titled “288 ×Vina”. The average across the four datasets is 1.83 Å, and only 30% of complexes could not be reproduced.

### DINC

In its current implementation, DINC can be seen as an incremental Multi-Vina approach (see “Methods” section). Therefore, we wanted to examine whether this additional incremental paradigm would give DINC an advantage over the regular Multi-Vina approach. For that, we ran five replicates of a redocking experiment involving all the complexes from our first four datasets. Results (averaged over the five replicates) are listed in Tables 1–4, under the column titled “DINC”. The overall average and standard deviation across the four datasets are 2.34 Å and 0.32 Å, respectively. When comparing these results to those obtained with the 24 ×Vina protocol, one can conclude that DINC seems to perform better than the Multi-Vina approach. Indeed, the improvement observed between 24 ×Vina and DINC is much larger than the improvement observed between the 12 ×Vina and 24 ×Vina protocols (see Fig. 1). This comparison is meaningful because, in terms of computing resources, DINC lies between a 24 ×Vina and a 36 ×Vina protocol.

To assess the full potential of DINC, we collected all the results from all the redocking experiments we performed when comparing the various DINC protocols (see “Methods” section). More specifically, for each complex of our first four datasets, we looked for the minimum RMSD among all the top-RMSD conformations generated by the replicates of the 15 DINC protocols we had evaluated (see “Methods” section). This amounts to running a Multi-DINC, similar to the Multi-Vina, except that different instances of DINC may use different parameters. Note that all DINC protocols run 12 Vina instances, except the final one, which runs 24 Vina_4_ instances (i.e., Vina with its exhaustiveness set to 4) as explained in the “Methods” section. The results we obtained by combining all the DINC protocols together are listed in Tables 1–4, under the column titled “DINC _*best*_”. The average across the four datasets is 1.55 Å, and only 17% of complexes could not be reproduced. Note that the amount of computing resources involved in obtaining the results reported for DINC _*best*_ is certainly greater than that used by the 288 ×Vina protocol, although a direct comparison is not really possible. Despite this fact, we can conclude that DINC shows a greater potential to reproduce challenging complexes than the Multi-Vina approach.

### PPDbench dataset

As the PPDbench dataset was published after we had performed our evaluation study [28], we used it only in a smaller experiment to compare the sampling capabilities of Vina and DINC’s default protocols (see “Methods” section). We performed five replicates of a redocking experiment in which we tried to reproduce the crystal structures of the 89 complexes of this dataset. The results we obtained are presented in Table 5. Vina could not reproduce any of these complexes. The average all-atom RMSD (across the whole dataset) between the initial crystal structure and the top-RMSD conformation produced by Vina is 7.7 Å with a standard deviation of 1.01 Å. Although DINC was able to successfully reproduce only 7 complexes, the average all-atom RMSD it achieved across the whole dataset is 4.17 Å, with a standard deviation of 0.45 Å. This represents a significant improvement in comparison to Vina. Results obtained on this dataset, which contains very large peptides (with up to 67 DoFs), illustrate that, even when using DINC, more sampling is required to reproduce such challenging complexes.

**Fig. 2:**
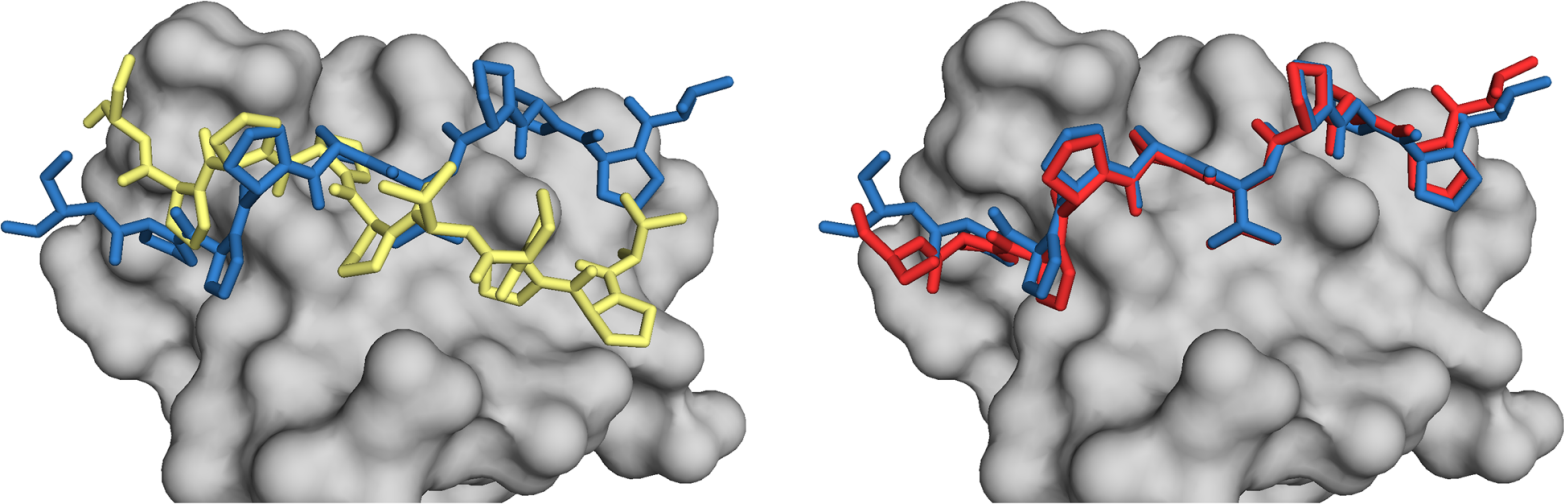
Binding modes predicted by Vina and DINC for the protein-peptide complex with PDB code 2O9V. The protein receptor is represented by a grey *surface* in both images. The conformation of the peptide ligand as reported in the 2O9V PDB entry is represented by blue *sticks* in both images. The best result obtained when redocking this peptide with Vina is represented by yellow *sticks* in the left-hand side image; the all-atom RMSD between this conformation and the blue one is 5.61 Å. The best result obtained when redocking this peptide with DINC is represented by red *sticks* in the right-hand side image; the all-atom RMSD between this conformation and the blue one is 1.1 Å; only the ends of the peptide are not well aligned with the crystal structure

To illustrate differences between a successful and unsuccessful reproduction of a specific complex, we report the best results obtained with Vina and DINC when trying to reproduce the protein-peptide complex with PDB code 2O9V (see Fig. 2). The peptide involved in this complex is composed of 69 heavy atoms and features 15 DoFs. The all-atom RMSD between the crystal structure and the top-RMSD conformations produced by Vina and DINC are 5.61 Å and 1.1 Å, respectively. As the threshold for success is 2 Å, the binding mode obtained with DINC constitutes a successful reproduction of the crystal structure. Figure 2 shows that only the ends of the peptide’s conformation are not very well aligned with the crystal structure. On the other hand, the binding mode produced by Vina corresponds to a totally different conformation.

## Discussion

### Vina’s exhaustiveness

The default value of Vina’s exhaustiveness is 8. When we increased its value to 100, we obtained better docking results for many protein-ligand complexes, but not all of them. One can mostly interpret the effect of raising exhaustiveness as increasing the amount of sampling performed by Vina during a given run. Therefore, the fact that increasing Vina’s exhaustiveness can sometimes lead to a deterioration of the docking results might be counter-intuitive. However, another effect of increased exhaustiveness is an enhanced impact of the scoring function on the final output, as Vina can spend more time improving the fit of the population of binding modes it internally maintains. In other words, raising Vina’s exhaustiveness increases the bias of its scoring function on the sampling procedure. If Vina’s scoring function was perfectly accurate, raising exhaustiveness would systematically yield better docking results. Unfortunately, as it is not perfect, Vina’s scoring function sometimes drives the sampling process away from near-native binding modes of a complex. In other cases the scoring function simply favors an alternative binding mode that is as valid as the one captured by the crystal structure.

Note that two docking studies focused on protein-peptide complexes have shown that, despite leading to huge increases in running times, raising exhaustiveness to large values does not produce drastic improvements [26, 27]. Therefore, increasing Vina’s exhaustiveness is clearly not the most effective use of computing resources when trying to improve docking results. In addition, our results suggest that in the context of a meta-docking approach involving several instances of Vina such as Multi-Vina or DINC, reducing exhaustiveness is beneficial. The first benefit is that it reduces running times. The second benefit is that it ensures more diversity in the sets of binding modes produced by all Vina instances, as the bias from the scoring function is reduced.

Vina’s exhaustiveness controls the number of independent runs that it performs internally. However, other parameters might have an impact on the achieved amount of sampling, such as the number of steps performed by each run, the length of the local optimization within each step, or the optimization method itself (knowing that Vina uses the Broyden-Fletcher-Goldfarb-Shanno algorithm). Unfortunately, the only parameter accessible to Vina’s users is the exhaustiveness.

The default protocol we have adopted in DINC uses Vina_4_, i.e., it runs Vina with its exhaustiveness set to 4. This allows reducing Vina’s running time by half, as compared to its regular version. In addition, after performing redocking experiments with DINC using either Vina_4_ or the regular Vina, we observed no significant change in results quality in terms of all-atom RMSD to the initial crystal structures. For very few complexes, using Vina_4_ improved docking results, and for even fewer complexes, using Vina_4_ deteriorated docking results. On the other hand, using Vina_2_ clearly deteriorated docking results on the four datasets. Therefore, using Vina_4_ seems to be a good compromise between achieving computational efficiency and obtaining docking results of good quality.

### Parallelized meta-docking

As discussed in the “Results” section, running several Vina instances in parallel and grouping their results together is a more efficient way to improve docking results than simply increasing exhaustiveness in a single instance of Vina. First, this kind of parallelized meta-docking approach is computationally efficient: even if it uses the same amount of computing resources as a long Vina run, it is much faster in terms of wall clock time. Second, with a given computing budget, the multi-threaded approach provides better docking results than the single-threaded approach.

This result is most likely not specific to Vina: a parallelized meta-docking approach using another docking tool would probably provide similar benefits. This was demonstrated, to some extent, by the original implementation of DINC [11, 17], which involved AutoDock4. Therefore, to rephrase the above statement in more general terms, it is more effective to combine the results from several short docking runs than extending a single docking run. This concept should be familiar to readers of the computational biophysics literature: better results have been obtained from combining several short molecular dynamics simulations together than running a very long simulation [29]. This paradigm has been applied in numerous fields of computer science, such as genetic programming [30].

### Parallelized incremental meta-docking

Despite achieving rather good results, the Multi-Vina approach failed at reproducing some complexes, even when using a very large number of threads. On the other hand, DINC was able to reproduce some of these complexes. In addition, when using a similar computing budget, DINC provides better docking results than the Multi-Vina approach. Therefore, the addition of the incremental paradigm to a simple parallelized meta-docking approach can be considered beneficial. Our understanding is that this is especially true when the binding site is not at the protein’s surface but deeper in the protein’s core. Complexes exhibiting this characteristic are often the results of significant conformational changes undergone by the protein receptor as a result of the docking process in vivo [6]. Therefore, reproducing their crystal structure might be impossible if one keeps the protein receptor rigid while attempting to dock the ligand in the binding site. More specifically, because the binding site is so constrained, it becomes difficult to computationally sample conformations of the whole ligand within it. On the other hand, docking a smaller fragment of this ligand and growing it in the binding site can be easier.

After evaluating several docking protocols in DINC, we had to conclude that none of them systematically performed best. Compared to the others, each protocol improves the docking results for some complexes and deteriorates them for other complexes. Therefore, we chose as a default protocol for our latest version of DINC the one providing a good trade-off between docking accuracy and computational efficiency. This protocol involves three rounds of incremental docking using Vina_4_; it was evaluated with 24 threads. To obtain better docking results, one can simply increase the number of threads. In addition, if enough computing resources are available, one can easily implement a Multi-DINC method in which the various DINC instances would use different protocols. The beauty of such a meta-docking strategy is that it can be implemented with as many levels as computing resources permit. As illustrated by our results, in this way, most protein-ligand complexes, even challenging ones involving large ligands, can be reproduced.

### Additional conformational sampling

The docking protocols we have presented can be combined with other techniques providing additional conformational sampling of a protein-ligand complex. For example, one could envision exploring the conformational space around binding modes produced by a docking protocol using molecular dynamics (MD) simulations. As simulating binding with MD is very expensive, MD has mostly been used for post-docking relaxation [31–34] or ensemble docking [35–37]. To ensure broader sampling of MD simulations, several strategies have been proposed, such as enhanced sampling (e.g., umbrella sampling, metadynamics, replica exchange) [38] or accelerated MD [39, 40].

## Conclusions

In this study, we have assessed the sampling power of several docking protocols involving the popular molecular docking tool Vina. More specifically, we have evaluated Vina with increased exhaustiveness, a protocol involving several instances of Vina running in parallel, called Multi-Vina, and our parallelized incremental meta-docking approach using Vina, called DINC. For this evaluation we have performed redocking experiments, trying to reproduce crystal structures of challenging protein-ligand complexes with large ligands. The five datasets we have used come from similar studies and contain complexes that classical docking tools could not reproduce. To try and separate as much as possible the sampling challenge from the scoring challenge, our assessment of docking results was based on evaluating the all-atom RMSD between the original crystal structure of a complex and the top-RMSD conformation generated by a docking approach for this complex. The rationale was to assess whether a docking approach could produce binding modes that were close enough to a native conformation, irrespective of whether the scoring function could select these binding modes as being the most favorable ones.

Our results show that increasing Vina’s exhaustiveness yields limited improvement, with few complexes being reproduced using this approach. Therefore, when dealing with large ligands, the increase in computing costs incurred from raising Vina’s exhaustiveness is not worthy of this small improvement in docking accuracy. Running several short Vina instances and grouping their results together in a Multi-Vina approach seems to be a better use of additional computing resources. Indeed, using this approach yields significant improvement in docking accuracy, with numerous complexes being successfully reproduced. However, even when using a very large number of threads, about a third of complexes still remain too challenging for the Multi-Vina approach. On the other hand, our incremental meta-docking approach, DINC, can successfully reproduce the vast majority of complexes studied here, albeit only when using a huge amount of computing resources for some of these complexes. In general, even when using a more reasonable amount of computing resources, DINC performs significantly better than the Multi-Vina approach, given a similar computing budget.

In conclusion, this study clearly demonstrates the benefits of using parallelized docking approaches, as well as incremental docking approaches such as our meta-docking tool DINC, to solve the sampling challenge associated with the docking of large ligands, including peptides. More generally, our results illustrate that conformational sampling is not really a challenge anymore, contrary to what transpires from previous similar studies [25–27]. The real challenge of molecular docking resides on improving scoring functions. In fact, methods such as Multi-Vina or DINC incur additional computing costs required to counter the bias imposed by the scoring function on the sampling procedure. For now, the solution we suggest is to use a meta-docking approach to generate a large pool of binding modes by grouping the results from several independent docking runs. The benefit is that this pool of binding modes can then be re-scored, using a scoring function that is more computationally-expensive but more accurate than the fast functions typically used by protein-ligand docking tools. We are planning to evaluate such re-scoring techniques in future work, as well as the consensus scoring paradigm [41].

## Methods

### DINC - docking incrementally

**Fig. 3:**
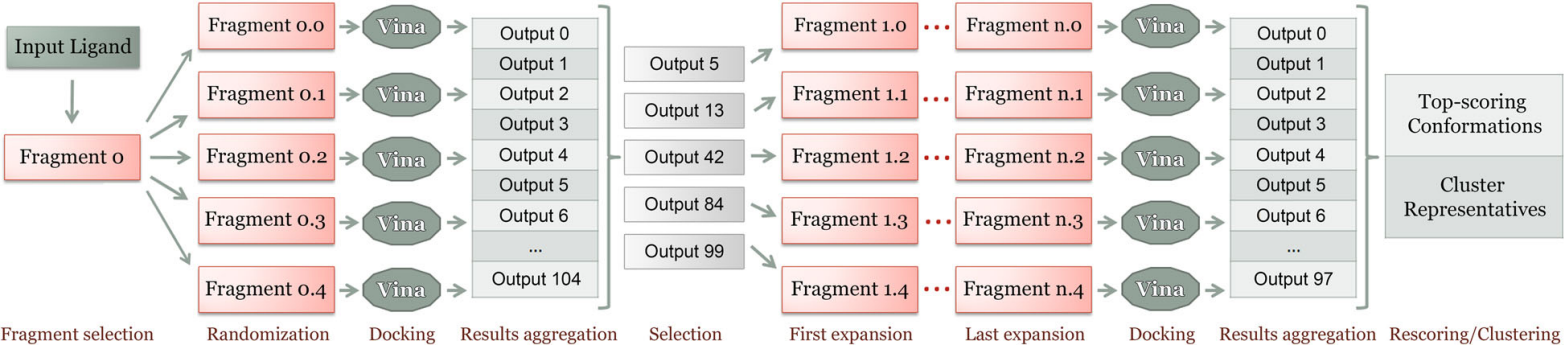
Workflow of the DINC algorithm on a specific example. DINC starts by selecting a small fragment of the input ligand (Fragment 0), with only *k* flexible bonds, and uses it as input for the first round of docking with Vina. The best binding modes are selected for expansion: they are “grown” by adding a small number of atoms. These new fragments are then docked in parallel using Vina. The process is repeated incrementally, until the entire input ligand has been reconstructed and is docked in the binding site

DINC is a parallelized meta-docking method developed for the incremental docking of large ligands to protein receptors. The rationale for the method and its implementation have been described in previous publications [11, 17, 20]. The newest version of DINC, called DINC 2.0, has been made available online as a web server [23, 24]. In short, DINC is based on a divide-and-conquer approach enabling the docking of large ligands containing too many flexible bonds to be efficiently docked by traditional protein-ligand docking tools. The idea behind DINC is to incrementally dock larger and larger overlapping fragments of a ligand instead of docking it all at once. The workflow of the algorithm is illustrated in Fig. 3.

Given a ligand, DINC’s algorithm starts by selecting a subset of the ligand’s flexible bonds to be explored, executing the sampling and scoring of this first fragment. Then, several docked conformations of this fragment are selected for expansion. During this process, the selected conformations are “grown” so as to include an additional subset of flexible bonds from the original ligand. This is defined by one of DINC’s parameters, which determines how many new flexible bonds are added at each round of docking. The expanded fragments are then used as input for a second round of sampling and scoring. This process is incrementally repeated until all the flexible bonds of the original ligand have been explored.

The number of flexible bonds that are explored in each round of docking is a key parameter for the success of the incremental process. Although the number of atoms and bonds composing the fragments increases from one round to the next, the number of bonds that are considered flexible and are effectively sampled is kept constant. In DINC, this parameter is referred to as the fragment size. Instead of defining the fragment size and the number of new flexible bonds (which then automatically determines the number of docking rounds), it is possible to define the number of docking rounds and new flexible bonds in DINC (which then automatically determines the fragment size). Note that different heuristics can be used to decide which bonds will be active at each round, and which previously-explored bonds will be kept rigid. The important point is that, by keeping only a subset of bonds active at each round, DINC enables the efficient sampling and scoring of the growing fragments.

Another key aspect of the DINC approach is parallelism. At each round of the incremental process, multiple attempts at docking a given fragment are performed independently in parallel. Then, all generated conformations are grouped together, and a subset of this conformation pool is selected for expansion and for the next docking round. In DINC, the parameter driving this behavior is referred to as the number of docking tasks, or simply the number of threads. Through parallelism, the amount of sampling at every round is greatly increased without affecting the overall running time. DINC can run on a desktop computer using multiple threads, and is also well-suited for high-performance computing systems.

DINC is also a meta-docking approach, in the sense that it relies on regular docking tools to perform the sampling and scoring tasks at each docking round. DINC itself only manages the parallelism, the generation of the fragments and the selection of active flexible bonds. In its original version, DINC relied solely on AutoDock4 [22]. The newest version of DINC, which we evaluate in this study, involves the popular docking tool Vina [21]. As two docking tools are available in DINC, it is now possible to use separate tools for sampling and scoring tasks. More generally, DINC is a highly customizable tool in which all parameters can be tuned.

### Docking protocols

The first docking protocol we evaluated involves only Vina and was aimed at studying the effects of varying Vina’s exhaustiveness. This parameter defines how long Vina will run by setting the number of independent runs that are performed internally, starting from random conformations of the ligand; its default value is 8. We increased it to 100, in a docking protocol that we refer to as Vina_100_, to evaluate whether this would improve Vina’s performance. While varying Vina’s exhaustiveness we kept its other parameters constant, but we did not use their default value. For the *num_modes* parameter, which defines the maximum number of binding modes that Vina can produce, we used a value of 25 instead of 9. For the *energy_range* parameter, which defines the maximum energy difference (in kcal/mol) allowed between the best and worst binding modes produced by Vina, we used a value of 10 instead of 3. Increasing the energy range and the maximum number of binding modes returned by Vina enabled us to rely less on its scoring function when analyzing its output.

We call the second docking protocol we evaluated Multi-Vina. It consists of running several independent instances of Vina in parallel and grouping their results together. This allows obtaining a larger set of binding modes that can be analyzed to evaluate the success of docking. In this context, we set Vina’s exhaustiveness to 8; we kept its num_modes and energy_range parameters at 25 and 10, respectively. We varied the number of Vina instances from 12 to 24, to 288 (=12×24), in docking protocols that we refer to as 12 ×Vina, 24 ×Vina and 288 ×Vina, respectively.

We then evaluated our parallelized incremental meta-docking tool, DINC. As DINC involves several parameters defining its incremental process, we wanted to assess which set of parameter values (i.e., which DINC protocol) would produce the best results. First, we varied the fragment size using the values 6, 12, 18, 24 and 30, while keeping all other parameters fixed: 12 threads and 3 new bonds at each docking round. Unfortunately, no fragment size value seemed to systematically produce the best results. Then, instead of using a fixed fragment size (which would result in a varying number of docking rounds based on the ligand’s number of flexible bonds) we decided to evaluate DINC protocols in which the number of docking rounds was fixed (therefore making the fragment size vary depending on the ligand). As number of docking rounds, we used the values 2, 3 and 4. For each value, we varied the number of new bonds at each docking round from 1 to 2 to 3. This resulted in 9 DINC protocols, each one running 12 threads. Overall, the protocol involving 3 rounds of docking with 3 new bonds at each round seemed to be performing best. However, better results were obtained for numerous complexes using other protocols. We do not present all these results here. We only report results achieved by DINC using what has now become our default protocol: 3 rounds of docking, with 3 new bonds, using 24 threads. Note that in all the DINC protocols we mentioned, we kept Vina’s num_modes and energy_range parameters at 25 and 10, respectively. On the other hand, we varied its exhaustiveness: reducing it from 8 to 4 did not seem to affect the docking results, contrary to reducing it to 2, which decreased docking accuracy. Therefore, to be more computationally efficient, the default DINC protocol now involves Vina_4_, i.e., Vina with its exhaustiveness set to 4.

### Evaluation methodology

To evaluate all the docking protocols we perform redocking experiments, in which we try to reproduce the crystal structure of protein-ligand complexes obtained from the Protein Data Bank (PDB) [42]. This requires processing the PDB files, following a standard procedure in the docking field: 1) removing water molecules; 2) if several instances of the complex are present in the PDB file, conserving only the first instance; 3) for each complex, separating the ligand from the protein and saving both molecules in separate PDB files that can be given as input to a docking tool. The preparation of the ligand and protein receptor is done using the scripts *prepare_ligand4* and *prepare_receptor4* from the AutoDockTools4 suite [22]. The operations performed by these scripts are: removing lone atoms, adding polar hydrogens, removing non-polar hydrogens, and adding Gasteiger charges. At the beginning of the docking process performed by DINC, similar to many other docking tools, the conformation of the given ligand is randomized. The objective of the redocking experiment is to assess whether the docking tool can produce a binding mode for the ligand in the protein’s binding site that is similar to the initial crystal structure.

The similarity between two binding modes of a protein-ligand complex (whether a computationally-generated binding mode or a crystal structure) is usually assessed by calculating the Root Mean Square Deviation (RMSD) between them. Several ways of calculating RMSD values have been reported in the molecular docking literature. Here, we use the strictest definition of the RMSD by calculating its values using all the heavy atoms of the ligand. This means measuring changes in the conformation, as well as in the position and orientation of the whole ligand within the protein’s binding site. As the protein is kept rigid and in a fixed position during the docking process, no alignment is required between two binding modes to calculate the RMSD between them. Note that a more lenient definition of the RMSD that is often used in related work consists of using only the backbone atoms of the ligand; in this case, changes in side-chain conformations are mostly ignored.

Similar to other docking tools, DINC produces several binding modes for a given protein-ligand complex as a result of the docking process. Therefore, there exists several ways to assess whether a docking tool was successful at reproducing a given crystal structure. If users decide to fully rely on their tool’s scoring function, they might decide to calculate the RMSD between the initial crystal structure and the binding mode that receives the best score, i.e., the so-called *top-scoring conformation*. A common threshold used to determine whether the crystal structure was successfully reproduced is 2 Å. Instead of considering only the top-scoring conformation, users can also check whether any of a small number of binding modes among those with the best scores have an RMSD of less than 2 Å from the crystal structure. In this study, as we want to evaluate the sampling power of the docking protocols, we calculate the RMSD between the crystal structure and all the produced binding modes, irrespective of their score, and we determine which one is the closest to the crystal structure, i.e., the so-called *top-RMSD conformation*. We consider that a given crystal structure has been successfully reproduced if the top-RMSD conformation is less than 2 Å away from it. This is common practice in the molecular docking field, which allows evaluating the sampling power of a docking tool independently (to some extent) of its scoring power.

### Datasets of protein-ligand complexes

For this study, we define five datasets containing protein-ligand complexes involving large ligands, some being peptides. These datasets comprise complexes involved in previous evaluations of molecular docking tools performed by several research groups. We restrict our datasets to complexes that some of these tools cannot reproduce. Our goal is to perform an indirect comparison between these tools and our own docking tool, DINC, by showing that it can reproduce most of these complexes.

**Dhanik dataset.**Our first dataset is a small subset of the one on which the original version of DINC was evaluated [11, 17]. The complete dataset contains 73 protein-ligand complexes extracted from an old version of the PDBbind database [43]. Results in [11, 17] show that DINC and AutoDock4 could reproduce the crystal structures of only 31 of these complexes, when considering top-RMSD conformations. The remaining 42 complexes could have thus been considered challenging. However, after running redocking experiments with the most recent version of Vina (using its default parameters), we realized that some of these complexes were actually not very challenging. Therefore, we removed from the dataset all the complexes that Vina could reproduce, when considering top-RMSD conformations. We also discarded the complexes featuring more than one ligand in the protein’s binding site. Eventually, we obtained a dataset containing 19 complexes, with ligands having between 7 and 30 rotatable bonds (see Table 1).

**Table 1:** Results of the redocking experiments performed on the Dhanik dataset

PDB ID	DoFs	atoms	Vina	Vina_100_	12 ×Vina	24 ×Vina	DINC	288 ×Vina	DINC _*best*_
2FZC	7	18	3.51 ±0.45	3.54 ±0.20	2.80 ±0.31	2.77 ±0.24	2.76 ±0.24	2.24	2.24
1TYR	8	22	5.17 ±1.66	4.17 ±1.73	2.77 ±0.16	2.35 ±0.28	1.82 ±0.26	1.81	1.62
1V2O	10	30	3.53 ±0.07	3.27 ±0.22	2.65 ±0.23	2.58 ±0.19	2.39 ±0.18	2.17	2.17
2DRC	10	33	7.19 ±0.39	7.69 ±0.64	3.51 ±2.69	3.30 ±2.56	1.06 ±0.18	1.00	0.97
1NWL	11	31	5.31 ±1.08	5.19 ±1.82	3.03 ±0.61	2.23 ±0.50	1.49 ±0.11	1.61	1.18
1ELB	13	33	4.56 ±0.13	4.48 ±0.03	3.35 ±0.58	2.72 ±0.57	2.18 ±0.28	2.20	1.90
3GSS	18	39	5.29 ±1.09	4.96 ±0.70	3.15 ±1.12	2.73 ±0.97	3.10 ±0.20	1.74	1.61
1IS0	18	47	5.90 ±1.34	3.37 ±0.80	3.44 ±0.55	2.20 ±0.71	2.20 ±0.33	1.36	1.32
1A1B	19	39	3.03 ±0.28	3.18 ±0.47	2.40 ±0.40	1.97 ±0.53	1.97 ±0.41	1.27	1.27
1JQ9	20	47	7.99 ±2.20	9.51 ±0.21	5.41 ±1.39	5.48 ±1.77	2.90 ±0.15	2.46	2.46
4FIV	20	58	2.77 ±1.28	1.11 ±0.50	0.79 ±0.36	0.60 ±0.03	0.62 ±0.09	0.55	0.49
4ER2	22	48	3.42 ±0.45	2.40 ±0.75	2.30 ±0.60	1.98 ±0.49	1.55 ±0.09	1.35	1.33
1G7Q	22	57	3.99 ±1.35	2.01 ±1.15	2.23 ±1.01	1.48 ±0.20	1.10 ±0.07	1.11	0.98
1SLG	23	59	7.30 ±1.79	6.43 ±2.17	3.76 ±1.42	3.13 ±1.01	2.36 ±0.10	2.13	1.91
1FZK	23	68	6.64 ±0.72	3.94 ±1.84	4.26 ±1.76	1.94 ±0.62	1.20 ±0.15	1.37	1.11
2ER9	25	65	2.87 ±0.39	2.67 ±0.29	2.09 ±0.21	2.10 ±0.34	1.87 ±0.11	1.52	1.52
1FKN	29	63	4.67 ±1.27	3.56 ±0.38	3.08 ±0.14	2.83 ±0.22	2.11 ±0.28	2.08	1.75
1PZ5	29	67	5.54 ±0.81	6.28 ±0.10	4.69 ±0.73	4.49 ±0.22	3.16 ±0.29	3.90	1.87
1FO0	30	70	7.36 ±1.22	4.78 ±1.08	3.29 ±1.83	2.92 ±1.57	1.05 ±0.19	1.04	0.73
average			5.05 ±0.95	4.35 ±0.79	3.11 ±0.85	2.62 ±0.69	1.94 ±0.20	1.73	1.50

For each complex in the dataset, we first list its identifier in the protein data bank (PDB ID), its number of rotatable bonds, i.e., degrees of freedom (DoFs), and its number of heavy atoms. Then, we list the average all-atom RMSD (over five replicates) associated with the top-RMSD conformation produced by Vina, Vina_100_, 12 ×Vina, 24 ×Vina, and DINC (see the “Results” section for explanations on these docking protocols), as well as the standard deviation. Finally, we list the all-atom RMSD associated with the top-RMSD conformation produced by 288 ×Vina and DINC _*best*_

**Renard dataset.**Our second dataset is a subset of a meta-dataset compiled from previous studies with the objective of assessing the ability of Vina to dock small peptides [27]. The original meta-dataset contains 47 complexes involving peptides with up to five amino acid residues. Results in [27] show that Vina could produce top-RMSD conformations that were similar to the crystal structure of only about half of these complexes. However, since these results were based on RMSD values calculated only for backbone atoms of the peptides, we performed our own redocking experiments to evaluate which complexes were really challenging. After discarding the complexes that already belonged to our first dataset, we were left with a set of 26 complexes (involving peptides having between 10 and 22 rotatable bonds) that Vina was not able to reproduce, even when considering top-RMSD conformations (see Table 2).

**Table 2:** Results of the redocking experiments performed on the Renard dataset. Description as in Table 1

PDB ID	DoFs	atoms	Vina	Vina_100_	12 ×Vina	24 ×Vina	DINC	288 ×Vina	DINC _*best*_
2FIB	10	30	2.03 ±0.12	1.34 ±0.24	1.19 ±0.39	1.31 ±0.45	0.81 ±0.13	0.70	0.70
2PQ2	11	26	4.45 ±0.63	4.20 ±0.59	3.52 ±0.61	3.12 ±0.06	3.02 ±0.08	2.66	1.76
1SUA	11	27	2.88 ±0.92	1.85 ±0.29	1.90 ±0.25	1.74 ±0.13	0.98 ±0.10	1.20	0.96
1NVR	12	29	3.24 ±1.02	3.17 ±1.11	1.14 ±0.16	1.20 ±0.19	1.20 ±0.18	0.74	0.74
2FNX	14	29	5.04 ±0.31	4.74 ±0.39	3.92 ±0.36	2.89 ±0.63	2.83 ±0.25	1.60	1.60
1TJ9	14	30	3.66 ±1.17	5.73 ±0.24	2.32 ±0.18	2.13 ±0.13	2.02 ±0.10	1.53	1.53
2DQK	14	33	4.24 ±0.44	3.92 ±0.16	3.51 ±0.34	3.41 ±0.39	2.42 ±0.37	2.00	2.00
1GYB	14	36	2.96 ±1.34	4.22 ±0.39	1.74 ±0.18	1.64 ±0.85	1.02 ±0.15	0.81	0.81
1TK4	15	30	6.66 ±1.25	7.70 ±0.39	4.83 ±0.54	3.46 ±0.92	2.87 ±0.75	2.03	1.78
1NX0	15	32	4.20 ±0.86	3.61 ±0.22	3.45 ±0.03	3.26 ±0.09	3.05 ±0.35	1.21	1.21
1BE9	15	35	4.70 ±1.84	3.41 ±1.86	1.38 ±0.23	1.21 ±0.27	1.25 ±0.23	0.85	0.85
1PAU	16	35	3.22 ±0.10	1.68 ±0.13	1.46 ±0.13	1.47 ±0.17	1.22 ±0.10	1.14	1.01
1IHJ	16	39	4.14 ±1.14	4.77 ±0.42	2.43 ±0.69	2.55 ±0.66	1.83 ±0.25	1.49	1.49
1JQ8	16	40	7.05 ±1.70	6.31 ±0.80	4.55 ±0.63	4.22 ±0.13	1.97 ±0.12	1.89	1.68
2HPL	17	41	3.35 ±0.67	2.60 ±0.40	1.78 ±0.34	1.51 ±0.36	1.52 ±0.35	0.98	0.98
2GNS	18	41	2.53 ±1.17	2.09 ±0.17	1.98 ±0.07	1.64 ±0.30	1.58 ±0.20	1.15	1.15
2D5W	19	37	4.78 ±2.61	1.43 ±0.10	1.38 ±0.08	1.33 ±0.06	1.16 ±0.12	1.14	1.14
1TJK	19	41	8.50 ±0.91	9.35 ±0.72	7.31 ±0.15	6.79 ±1.09	4.28 ±0.15	1.22	1.22
1JWG	19	43	4.95 ±0.51	6.03 ±1.12	3.38 ±0.75	3.38 ±0.76	2.43 ±0.08	2.16	1.78
2DUJ	19	43	3.40 ±0.51	2.99 ±0.27	2.21 ±0.34	2.28 ±0.14	2.24 ±0.06	1.65	1.65
1TG4	19	46	8.97 ±0.37	8.66 ±0.20	7.82 ±0.25	7.78 ±0.11	3.50 ±0.79	0.77	0.77
1SP5	20	46	4.23 ±1.13	5.29 ±0.16	1.69 ±0.65	1.34 ±0.13	1.14 ±0.07	1.01	1.01
1W9E	20	52	5.91 ±1.61	4.59 ±2.54	3.19 ±1.29	2.23 ±0.95	2.32 ±0.54	1.54	1.54
1FCH	21	45	3.82 ±1.32	5.00 ±1.76	1.93 ±0.35	2.03 ±0.60	1.85 ±0.45	1.07	1.07
1BHX	21	46	4.47 ±0.52	4.38 ±0.85	3.36 ±0.66	2.60 ±0.35	2.86 ±0.56	1.33	1.33
2H9M	22	41	4.05 ±0.99	3.33 ±0.62	2.81 ±0.24	2.25 ±0.65	2.46 ±0.03	1.43	1.35
average			4.52 ±0.97	4.32 ±0.62	2.93 ±0.38	2.64 ±0.41	2.07 ±0.25	1.36	1.27

**LEADS dataset.**Our third dataset is a subset of LEADS-PEP, which currently contains 53 protein-peptide complexes involving peptides composed of 3 to 12 residues [26]. LEADS-PEP was created as an unbiased benchmark dataset for researchers wanting to assess the efficacy of molecular docking tools on peptides. It was used to evaluate four protein-ligand docking tools: GOLD, Surflex-Dock, AutoDock4 and Vina [26]. Results show that, in spite of not being specifically aimed at peptides, these four tools were able to perform quite well with small peptides composed of 3 or 4 residues. However, they all showed poor performance on larger peptides, even when considering top-RMSD conformations, and despite the fact that reported RMSD values were calculated using only backbone atoms. Therefore, we removed from the dataset complexes containing small peptides, as well as complexes already present in previous datasets. The resulting dataset contains 33 complexes involving peptides having between 11 and 52 rotatable bonds (see Table 3).

**Table 3:** Results of the redocking experiments performed on the LEADS dataset. Description as in Table 1

PDB ID	DoFs	atoms	Vina	Vina_100_	12 ×Vina	24 ×Vina	DINC	288 ×Vina	DINC _*best*_
1UOP	11	32	2.42 ±1.56	1.61 ±0.14	1.07 ±0.10	1.01 ±0.12	1.09 ±0.14	0.62	0.62
2HPL	17	41	2.41 ±0.26	2.41 ±0.43	1.73 ±0.21	1.40 ±0.11	1.62 ±0.33	1.08	1.08
4V3I	18	41	5.44 ±0.67	4.68 ±0.18	4.28 ±0.36	3.58 ±0.90	3.90 ±1.03	2.00	2.00
3OBQ	19	62	4.91 ±2.12	7.87 ±0.47	4.33 ±0.73	4.08 ±0.91	2.67 ±1.02	1.29	1.29
3LNY	20	44	4.26 ±1.31	7.44 ±4.81	2.04 ±0.16	1.88 ±0.07	1.86 ±0.06	1.37	1.37
3D1E	20	45	5.41 ±2.41	5.48 ±2.33	2.30 ±0.61	2.26 ±0.41	1.98 ±0.34	1.27	1.27
2W0Z	20	67	4.16 ±1.47	4.68 ±0.05	3.31 ±1.12	2.64 ±0.38	2.67 ±0.64	1.10	1.10
1SVZ	21	55	3.20 ±1.05	1.70 ±0.51	1.38 ±0.22	1.44 ±0.20	1.40 ±0.04	0.79	0.79
3Q47	22	53	2.75 ±1.55	1.13 ±0.16	1.01 ±0.12	0.95 ±0.09	0.92 ±0.08	0.76	0.76
3IDG	23	53	4.65 ±0.39	2.92 ±0.86	3.09 ±0.16	2.63 ±0.46	1.87 ±0.14	1.75	1.57
3UPV	24	55	4.56 ±0.69	3.64 ±1.57	1.88 ±0.56	1.36 ±0.27	2.33 ±0.80	1.14	1.14
3CH8	24	66	2.70 ±2.29	1.00 ±0.26	0.89 ±0.22	0.86 ±0.13	0.70 ±0.04	0.67	0.60
4Q6H	25	51	8.34 ±0.45	7.89 ±0.23	4.99 ±0.89	5.19 ±1.38	3.93 ±0.20	3.72	1.81
3NJG	26	58	5.01 ±2.77	1.46 ±0.16	1.25 ±0.13	1.25 ±0.09	1.09 ±0.18	1.07	0.99
1ELW	26	60	4.28 ±1.38	4.83 ±0.46	3.42 ±0.29	2.93 ±0.46	3.13 ±0.29	2.31	2.31
4QBR	27	53	2.72 ±0.53	2.42 ±0.44	1.50 ±0.26	1.51 ±0.20	1.37 ±0.16	1.16	1.16
1N7F	27	62	12.46 ±0.79	12.48 ±0.71	9.92 ±1.97	9.96 ±0.39	6.89 ±0.56	6.38	3.77
3MMG	29	60	5.28 ±2.93	1.72 ±0.37	1.58 ±0.35	1.86 ±0.29	1.16 ±0.21	1.16	1.16
2O02	32	68	4.98 ±0.70	5.06 ±0.73	4.32 ±0.65	4.36 ±0.34	2.83 ±0.25	3.18	1.97
3BRL	32	68	7.23 ±0.34	7.15 ±1.27	5.03 ±0.33	4.49 ±0.79	3.36 ±0.57	2.92	1.46
2W10	32	90	8.17 ±2.77	7.52 ±1.32	3.83 ±0.97	3.21 ±0.25	3.16 ±0.69	2.15	2.15
4EIK	34	86	4.06 ±0.96	1.94 ±0.53	1.75 ±0.44	1.81 ±0.25	1.35 ±0.22	0.98	0.98
4DS1	35	77	7.34 ±2.16	7.32 ±2.39	4.49 ±1.89	5.14 ±0.31	1.35 ±0.19	1.17	1.17
1NTV	39	89	5.84 ±0.82	5.67 ±1.22	4.03 ±1.08	4.07 ±0.21	3.92 ±0.58	1.85	1.85
1N12	40	85	10.04 ±2.88	2.75 ±1.21	4.39 ±1.65	3.77 ±1.72	4.05 ±1.57	1.84	1.44
2QAB	41	79	6.22 ±0.74	5.79 ±0.62	4.75 ±0.25	4.52 ±0.37	5.16 ±0.28	3.90	3.90
3DS1	41	95	6.19 ±0.12	5.94 ±0.48	5.15 ±0.32	4.34 ±1.37	3.65 ±1.02	1.73	1.73
1H6W	42	85	11.98 ±0.94	8.19 ±3.88	9.68 ±0.93	10.12 ±0.15	1.57 ±0.20	2.38	1.57
3BFW	43	89	6.52 ±1.72	2.19 ±1.44	2.98 ±0.89	2.64 ±1.18	3.49 ±1.06	1.59	1.41
2XFX	43	90	6.22 ±1.44	4.56 ±1.26	4.30 ±0.99	3.02 ±0.95	2.52 ±0.67	1.72	1.11
4DGY	44	98	6.88 ±0.90	4.96 ±1.13	3.27 ±1.09	4.29 ±0.39	3.39 ±0.96	1.93	1.93
4J8S	50	102	6.04 ±1.36	6.03 ±0.41	5.10 ±0.31	4.72 ±0.23	4.54 ±0.38	4.45	2.96
2B9H	52	101	8.20 ±3.14	8.49 ±2.58	5.39 ±0.48	4.70 ±0.35	4.13 ±0.40	3.56	3.56
average			5.78 ±1.38	4.82 ±1.05	3.59 ±0.63	3.39 ±0.48	2.70 ±0.46	1.97	1.64

**Hou dataset.**Our fourth dataset is a small subset of an extensive dataset used to evaluate ten molecular docking tools, including AutoDock4 and Vina [25]. The complete dataset contains 2002 protein-ligand complexes extracted from a recent version of the PDBbind database [44]. Results in [25] clearly show that all docking tools struggle with neutral ligands and large ligands, such as peptides. Interestingly, a set of 72 complexes could not be reproduced by any of the tested docking tools, even when considering top-RMSD conformations, and are, therefore, ideal candidates for our evaluation of challenging protein-ligand complexes. After discarding from this small set complexes involving more than one ligand in the binding site and complexes involving ligands with less than 7 rotatable bonds, we were left with a dataset containing 28 complexes involving ligands with 7 to 31 rotatable bonds (see Table 4).

**Table 4:** Results of the redocking experiments performed on the Hou dataset. Description as in Table 1

PDB ID	DoFs	atoms	Vina	Vina_100_	12 ×Vina	24 ×Vina	DINC	288 ×Vina	DINC _*best*_
2W5G	7	38	5.29 ±2.11	3.84 ±2.23	2.20 ±0.72	1.46 ±0.55	1.09 ±0.05	0.84	0.84
3EB1	8	30	5.60 ±0.16	5.72 ±0.03	4.68 ±0.44	4.52 ±0.17	3.01 ±0.57	4.23	1.96
2BVR	9	31	5.78 ±0.87	4.79 ±0.35	4.08 ±0.60	3.69 ±0.61	3.55 ±0.57	3.03	1.90
3AAQ	9	42	2.30 ±0.21	1.76 ±0.70	1.56 ±0.50	0.96 ±0.47	2.07 ±0.10	0.55	0.55
3UIL	10	14	7.78 ±0.59	8.78 ±0.58	6.22 ±0.29	6.30 ±0.15	2.79 ±0.21	3.05	2.56
3USX	12	16	6.78 ±0.25	6.92 ±0.17	5.84 ±0.42	5.45 ±0.61	2.89 ±0.84	2.72	2.05
1G7V	12	29	3.36 ±0.58	5.36 ±0.36	2.34 ±0.41	2.37 ±0.24	2.43 ±0.27	1.87	1.72
3DRI	13	42	8.13 ±0.91	8.51 ±1.17	5.46 ±1.09	4.15 ±0.37	3.46 ±0.45	3.90	2.94
3EAX	14	48	5.93 ±0.63	6.16 ±0.43	5.29 ±0.19	5.20 ±0.45	4.85 ±0.23	4.41	4.29
3R0Y	15	50	4.46 ±0.52	4.86 ±0.05	4.07 ±0.10	3.93 ±0.04	3.85 ±0.10	3.89	3.34
4FNN	16	20	3.87 ±1.01	4.23 ±0.53	2.46 ±0.49	2.31 ±0.40	1.75 ±0.23	1.42	1.42
2BVS	17	42	4.62 ±1.10	4.11 ±0.16	3.31 ±0.60	3.29 ±0.70	3.01 ±0.69	2.06	2.06
3FVH	18	53	4.87 ±0.20	1.46 ±0.20	4.21 ±0.10	3.61 ±0.44	3.07 ±0.29	2.90	2.23
1SH9	18	56	4.73 ±3.16	1.63 ±0.37	1.55 ±0.41	1.39 ±0.17	1.25 ±0.07	0.82	0.82
1STR	20	64	2.73 ±0.86	3.29 ±1.13	2.06 ±0.30	1.74 ±0.24	1.85 ±0.12	1.37	1.37
2CE9	20	68	5.46 ±1.76	6.96 ±0.26	2.70 ±2.05	2.46 ±1.72	1.28 ±0.15	0.86	0.86
3GX0	21	50	5.27 ±0.76	4.12 ±0.79	2.45 ±0.71	2.64 ±1.18	1.76 ±0.10	1.63	1.63
3JZH	21	54	7.13 ±0.31	8.23 ±0.89	6.57 ±0.13	6.61 ±0.12	5.15 ±1.53	4.93	2.48
4E67	22	68	2.54 ±0.86	2.62 ±0.18	1.08 ±0.30	1.12 ±0.21	1.24 ±0.25	0.63	0.63
3H89	23	81	5.18 ±0.91	5.81 ±0.10	3.46 ±0.29	4.00 ±0.98	1.64 ±0.31	2.71	1.33
2HKF	25	70	5.69 ±1.30	3.16 ±0.78	2.62 ±0.88	1.99 ±0.67	1.67 ±0.30	1.25	1.25
4GAH	27	71	6.17 ±2.24	3.34 ±0.74	3.22 ±0.81	2.92 ±0.66	2.08 ±0.24	1.51	1.51
3IFL	27	76	4.35 ±0.32	3.96 ±0.13	3.08 ±0.47	2.33 ±0.31	2.32 ±0.06	1.72	1.72
4EZX	28	72	5.56 ±0.94	6.20 ±0.18	5.27 ±0.68	5.29 ±0.18	3.32 ±0.58	3.29	2.23
3URI	28	79	5.17 ±1.22	2.58 ±0.91	2.15 ±0.28	1.84 ±0.30	1.76 ±0.15	1.45	1.45
1BAI	29	75	3.79 ±1.94	2.20 ±0.25	1.78 ±0.32	1.84 ±0.14	1.74 ±0.09	1.36	1.36
2ER6	30	78	5.75 ±1.84	1.58 ±0.06	2.20 ±0.81	1.65 ±0.16	1.50 ±0.11	1.26	1.26
1M4H	31	76	4.98 ±2.53	2.92 ±0.64	2.29 ±0.35	2.19 ±0.64	2.08 ±0.16	1.51	1.51
average			5.12 ±1.07	4.47 ±0.51	3.36 ±0.53	3.12 ±0.46	2.44 ±0.32	2.18	1.76

**Table 5:** Results of the redocking experiments performed on the PPDbench dataset

PDB ID	DoFs	atoms	Vina	DINC	PDB ID	DoFs	atoms	Vina	DINC
2HO2	13	71	7.58 ±1.30	2.64 ±0.41	4B4N	40	110	8.82 ±0.95	5.21 ±0.61
2O9V	15	69	6.07 ±0.29	1.98 ±0.77	3AWR	41	84	8.25 ±0.74	4.94 ±0.37
3OBQ	19	62	7.50 ±0.70	2.52 ±0.93	2PEH	41	88	7.13 ±1.10	4.54 ±0.15
1CKA	22	65	6.78 ±0.18	2.68 ±0.49	2XVC	41	99	10.67 ±1.56	2.53 ±0.35
2A25	23	60	10.65 ±1.54	1.45 ±0.98	3GYT	42	81	7.14 ±0.47	5.63 ±0.30
2R9Q	24	59	5.93 ±0.72	2.25 ±0.22	1H6W	42	85	11.13 ±2.14	2.65 ±1.68
4H4F	25	72	3.75 ±0.20	1.05 ±0.03	2O4J	42	87	8.18 ±0.95	4.82 ±0.56
1SSH	25	76	8.31 ±1.04	5.04 ±0.30	4GQ6	42	99	6.98 ±1.36	4.65 ±0.28
3TJV	26	63	5.45 ±1.78	1.43 ±0.05	2XRW	42	100	9.19 ±1.24	6.26 ±0.67
3ERY	27	70	6.26 ±1.55	3.10 ±0.38	3DS4	42	103	7.85 ±0.82	4.79 ±0.36
3I5R	27	70	5.71 ±0.24	3.67 ±0.63	2PUY	43	80	8.94 ±2.46	3.94 ±0.30
1MFG	29	71	7.27 ±1.78	3.88 ±0.13	2P1T	43	86	5.86 ±1.09	4.05 ±0.74
1YWO	29	80	6.89 ±1.66	3.50 ±0.23	1D4T	44	90	14.65 ±1.08	4.81 ±0.87
1QKZ	30	62	5.63 ±1.71	2.82 ±0.39	2QSE	45	89	7.21 ±0.68	5.25 ±0.23
1K5N	30	64	4.66 ±0.99	2.84 ±0.63	1RXZ	45	97	7.35 ±0.81	3.53 ±0.91
1OAI	30	66	7.75 ±0.28	3.03 ±0.43	2BBA	45	115	7.18 ±0.82	5.08 ±0.35
1U00	30	71	7.01 ±1.08	2.20 ±0.15	1NQ7	46	90	8.06 ±0.41	5.46 ±0.30
1RST	30	76	7.16 ±0.36	4.37 ±0.14	3KMR	46	90	7.02 ±0.86	5.36 ±0.36
4GXL	30	81	7.41 ±1.01	2.69 ±0.20	3VTC	46	91	8.59 ±0.91	5.63 ±0.11
3RM1	31	71	6.23 ±0.88	3.21 ±0.63	2P54	46	97	7.40 ±0.75	5.14 ±0.34
2D0N	32	70	9.69 ±2.48	3.56 ±0.54	2QOS	46	97	7.64 ±0.37	5.89 ±0.33
2VR3	32	71	12.06 ±2.67	6.30 ±1.43	3RQG	47	92	7.09 ±0.29	5.34 ±0.24
4HTP	32	85	9.02 ±0.27	4.52 ±0.88	4ERY	47	100	7.19 ±0.33	3.61 ±0.59
2CE8	33	70	7.19 ±0.35	4.59 ±0.66	2FTS	47	104	8.04 ±0.42	4.54 ±0.24
4F1Z	33	72	15.69 ±2.99	2.88 ±0.17	1OW6	48	94	9.95 ±0.52	7.23 ±0.39
2ZJD	33	77	2.77 ±0.53	1.79 ±0.07	3C3R	48	104	8.94 ±1.47	5.87 ±0.34
3W1B	33	85	9.93 ±0.52	5.21 ±1.19	3L0E	49	98	7.08 ±1.09	5.22 ±0.10
3V2X	34	84	10.65 ±0.96	2.56 ±0.46	3OLF	49	99	6.51 ±0.57	5.49 ±0.27
3PTL	35	71	10.92 ±2.82	3.99 ±0.19	1T08	49	115	8.98 ±0.81	5.36 ±0.97
1X2R	35	74	6.51 ±0.95	4.12 ±0.40	1NLN	50	94	7.41 ±0.88	2.96 ±0.85
3KUS	35	83	7.28 ±1.99	2.53 ±0.57	4J8S	50	102	6.63 ±0.61	4.79 ±0.18
3U9Q	36	69	5.64 ±1.24	3.34 ±0.35	2FMF	50	107	9.26 ±0.85	6.54 ±0.33
1T7R	36	77	5.21 ±0.30	3.15 ±0.47	2FFF	50	109	7.98 ±0.52	4.72 ±0.77
2QBX	36	80	4.02 ±0.80	2.47 ±0.56	3QIS	50	109	5.91 ±0.44	3.85 ±0.49
1NX1	36	81	5.57 ±0.65	4.01 ±0.47	2PUX	50	111	9.27 ±1.71	5.18 ±0.28
2W2U	36	91	6.17 ±1.49	3.49 ±0.49	2CCH	51	102	8.26 ±0.45	4.94 ±0.81
1UJ0	37	74	7.02 ±1.10	3.89 ±0.40	2WHX	51	109	8.41 ±0.69	4.98 ±0.72
2HT9	37	85	7.72 ±0.75	5.74 ±0.23	2VWF	51	115	8.28 ±1.55	5.65 ±0.22
1EG4	37	106	8.97 ±0.86	4.64 ±0.26	2B9H	52	101	9.73 ±2.17	4.53 ±0.53
2FVJ	38	81	6.83 ±0.44	5.13 ±0.24	3UP3	53	108	8.75 ±0.83	6.28 ±0.19
3LL8	38	85	3.70 ±1.01	1.98 ±0.15	3H1Z	53	136	9.89 ±2.02	5.04 ±0.33
1T4F	38	86	7.52 ±0.81	5.27 ±0.13	1PZL	55	114	7.79 ±0.62	5.62 ±0.31
1TFC	39	81	7.02 ±0.64	4.95 ±0.28	4K0U	62	130	8.31 ±0.74	5.90 ±0.49
2DYP	39	81	3.70 ±0.91	1.71 ±0.12	2V8Y	67	129	9.10 ±1.42	6.09 ±0.36
1NTV	39	89	6.20 ±0.87	3.27 ±0.69	average			7.7 ±1.01	4.17 ±0.45

For each complex in the dataset, we first list its identifier in the protein data bank (PDB ID), its number of rotatable bonds, i.e., degrees of freedom (DoFs), and its number of heavy atoms. Then, we list the average all-atom RMSD (over five replicates) associated with the top-RMSD conformation produced by Vina and DINC using their defaults parameters

**PPDbench dataset.**Our fifth dataset was published after we carried out our benchmarking. Therefore, we used it only for a smaller experiment to compare results obtained with Vina and DINC using their default parameters. The original PPDbench dataset was involved in a study comparing the performance of six molecular docking tools: ZDOCK, FRODOCK, Hex, PatchDock, ATTRACT and pepATTRACT [28]. It was created by combining two smaller datasets published in previous studies, and contains 133 protein-peptide complexes composed of 9 to 15 amino acids. After discarding from the original dataset the complexes involving more than one ligand in the binding site, we obtained a dataset containing 89 complexes involving peptides with 13 to 67 rotatable bonds (see Table 5).

### Evaluation platform

We ran all our docking jobs on the Comet cluster, from the San Diego Supercomputer Center, through an Extreme Science and Engineering Discovery Environment (XSEDE) allocation [45]. Comet features Intel next-gen processors with AVX2, Mellanox FDR InfiniBand interconnects and Aeon storage. Its compute nodes consist of Intel Xeon E5-2680v3 processors, 128 GB DDR4 DRAM, and 320 GB of SSD local scratch memory. Each node contains 24 cores, with a clock speed of 2.5 GHz, a flop speed of 960 GFlop/s and a memory bandwidth of 120 GB/s.

## Data Availability

The protein-ligand complexes contained in the five datasets used in this study are available in the Protein Data Bank (PDB) [42], through their PDB IDs listed in Tables 1-5. The code of our software, DINC, is available from the corresponding author upon request. DINC 2.0 is also available as a web server at http://dinc.kavrakilab.org.

## Abbreviations

DINC: Docking incrementally
DoF: Degree of freedom
MD: Molecular dynamics
PDB: Protein data bank
RMSD: Root mean square deviation
